# Association between the metabolic score for insulin resistance (METS-IR) and arterial stiffness among health check-up population in Japan: a retrospective cross-sectional study

**DOI:** 10.3389/fendo.2023.1308719

**Published:** 2024-01-02

**Authors:** Gailing Liu

**Affiliations:** Department of Nephrology, People’s Hospital of Zhengzhou University, He’nan Provincial People’s Hospital, He’nan Provincial Key Laboratory of Kidney Disease and Immunology, Zhengzhou, China

**Keywords:** metabolic score for insulin resistance (METS-IR), brachial-ankle pulse wave velocity (baPWV), insulin resistance, arterial stiffness, atherosclerosis

## Abstract

**Aim:**

This study examined the association between the metabolic score for insulin resistance (METS-IR), a novel surrogate indicator of insulin resistance, and brachial-ankle pulse wave velocity (baPWV) in Japanese health check participants.

**Methods:**

A cross-sectional research was conducted involving individuals in a program of medical health screening at the Medical Health Check-up Center in Japan. The study assessed the connection between METS-IR and baPWV in 912 participants who joined the program between 3/1/2004 and 12/31/2012. Serum laboratory tests and lifestyle surveys were the covariates. Multivariate linear regression analysis and subgroup analyses were performed.

**Results:**

912 participants were involved in this cross-sectional study. Adjusted for age, sex, BMI, AST, UA, HDL, eGFR, ankle-brachial index (ABI), alcohol consumption, and smoking status, multivariate linear regression analysis showed that METS-IR and baPWV showed a significant positive association (adjusted β=15.3, 95% confidence interval (CI): 6.61~23.98) with METS-IR as a continuous variable. When converting METS-IR to quartile categorical variables, higher quartile METS-IR indices had longer baPWV (Q3 vs Q1, β=86.14, 95% CI: 23.45~148.83; Q4 vs Q1, β=125.41, 95% CI: 39.99~210.84). In subgroup analysis, METS-IR was associated with baPWV in people with eGFR > 60 ml/min (adjusted β= 14.44, 95% CI: 5.61~23.26, P=0.001), none or light alcohol consumption (adjusted β=16.92, 95% CI: 6.85~27, P=0.001), non-smokers (adjusted β=15.48, 95% CI: 5.86~25.1, P=0.002), non-regular exercisers(adjusted β=17.34, 95% CI: 8.03~26.65, P<0.001), non-fatty liver (adjusted β=17.65, 95% CI: 5.92~29.39, P=0.003), and non-hypertensive (adjusted β=16.13, 95% CI:8.45~23.8, P<0.001).

**Conclusion:**

METS-IR are remarkably associated with baPWV among health check-up participants in Gifu, Japan. As a simple, easily calculated predictor of arterial stiffness, METS-IR could be considered for primary care as a monitoring tool to identify people with high risk of cardiovascular disease in order to intervene early on risk factors. Future prospective, large-sample researches are still needed to confirm this.

## 1 Introduction

Globally, cardiovascular illnesses continue to account for the greatest number of deaths and hospitalizations (1). Arterial stiffness is a powerful indicator of cardiac health, and it is linked to a higher risk of cardiovascular death (2–4). In clinical practice, it is crucial to identify patients who exhibit signs of subclinical cardiovascular system dysfunction in the general population, and PWV measurement is a useful tool for this (5, 6). The brachial-ankle pulse wave velocity (baPWV) is accomplished by applying pressure sleeves to the limbs and measuring the pulse wave velocity (7). According to the majority of studies, the increase in baPWV is associated with high blood pressure, diabetes, heart-brain vascular disease, and death (8).

Insulin resistance (IR) is known to exist in the early stages of numerous chronic diseases, including hypertension, metabolic syndrome, type 2 diabetes (DM), and chronic kidney disease (CKD) (9–11). The metabolic score for insulin resistance (METS-IR), a novel non-insulin-based index of fasting IR, has gained increasing attention in recent years (12, 13). Due to its non-invasive nature and its simple and convenient calculation, it has become more popular, particularly in epidemiological studies. Previous studies have shown that the metabolic syndrome promotes arterial stiffness, accelerates vascular aging and the development of hypertension in humans (6, 11, 14). Therefore, I hypothesized that there may be an association between IR or related surrogate markers and baPWV.

Some studies have demonstrated a correlation between some non-insulin-based IR indicators and arterial stiffness in populations such as hypertensive or elderly people (15–18). Triglyceride to HDL-cholesterol ratio (TG/HDL-C), triglyceride/glucose (TyG) index, and others are among these indicators. However, few studies have addressed the association of METS-IR with baPWV. Therefore, this cross-sectional research was constructed to see if there is a strong link between METS-IR and baPWV in the Japanese health check-up population.

## 2 Methods

### 2.1 Study design and population

Participants in Murakami Memorial Hospital’s Medical Health Checkup Center in Gifu, Japan, were studied in this cross-sectional study. This initiative detects and assesses chronic diseases to improve public health. This kind of “manual dock” medical checkup is popular among Japanese people.

Data was provided by DATADRYAD (http://www.Datadryad.org/). Free Dryad data packages are available to researchers (Fukuda, Takuya, et al., 2016) (19). Information about the work by Fukuda, Takuya, et al. was gathered using thorough citations (https://doi.org/10.5061/dryad.m484p).

All participants who had their baPWV measured during such health check-up programs at “Murakami Memorial Hospital” between March 2004 and December 2012 were recruited to participate in this research. Following the Declaration of Helsinki, the study was conducted. Every participant provided informed consent. This research adhered to the STROBE (Strengthening the Reporting of Observational Studies in Epidemiology) reporting guidelines (20).

Women who tested positive for hepatitis B antigen and/or hepatitis C antibody, such as those who were pregnant or taking oral contraceptives or hormone replacement treatment, were excluded from the study. To make sure the baPWV readings are accurate, subjects having an ankle-brachial index (ABI) of below 0.95 were excluded (19).

### 2.2 Data collection and measurements

The detailed data collection and measurement methods for this health screening program refer to the initial work by Fukuda T et al. The health check-up programs collected data using urinalysis, blood cell counts, blood chemistry, hepatitis B and C antigen and antibody measurements, the electrocardiogram (ECG), chest radiography, barium examination of the upper gastrointestinal tract, and abdominal ultrasonography (21). Expert abdominal ultrasonography with an Aloka SSD-650CL diagnosed fatty liver (Aloka Co., Ltd., Tokyo, Japan). One doctor examined all photocopied ultrasonographic photos and diagnosed fatty liver without consulting participants’ personal information (22). Hepatorenal echo contrast, liver brightness, deep attenuation, and vascular blurring were the four criteria for diagnosing fatty liver (23). Participants’ BMI was calculated by dividing their height in meters squared by their weight in kilos. This is how the METS-IR was determined: Ln [(2 × fasting glucose (mg/dL)) +fasting TG (mg/dL)]×BMI (kg/m2))/(Ln[high‐density lipoprotein cholesterol (mg/dL)]). eGFR was calculated as follows: eGFR=194×Cr−1.094×age−0.287 (mL/min/1.73 m2) for men, and eGFR was multiplied by a correction factor of 0.739 for women.

Automatic waveform analyzers measured baPWV and ABI (Colin Medical Technology, Komaki, Japan). After resting in a quiet room for 5 minutes, the subjects inserted ECG electrodes and a microphone for heart sounds on both wrists and the left sternal border. Brachial and ankle cuffs with plethysmographic and oscillometric sensors were worn. Thereafter, Fukuda T et al. (19) have figured out the lengths of the paths from the suprasternal notch to the brachium (Lb) and to the ankle (La). Finally, (La-Lb)/DTba calculates baPWV. The original study detailed how other covariates were measured and assessed (19).

The same trained interviewers gave each participant a standardized questionnaire. Researchers estimated weekly alcohol use by asking participants how many and what kind of alcoholic beverages they had the previous month. The total amount of alcohol used each week was measured in grams and categorized into four categories: nil or little use (40 g/week); light use (40–140 g/week); moderate use (140–280 g/week); and excessive use (>280 g/week) (22, 24, 25). Smoking status was classified into never, former, and current smoker (22). The survey asked about sports, leisure, and frequency (26). Regular exercisers participated in any sport at least once a week (27).

### 2.3 Statistical analysis

Categorical variables were shown by percentages, with continuous variables expressed as means (SD) or medians (IQR), depending on the situation. One-way analyses of variance (for normally distributed data), Kruskal-Wallis tests (for skewed data), and chi-square tests (for categorical data) were performed to examine the differences between the groups. Multivariate linear regression models were used to determine the effect values (β) and 95% confidence intervals (95% CIs) for the relationship between METS-IR and baPWV.

Three models were estimated: Model 1 was adjusted for age, sex and BMI. Model 2 was additionally adjusted for age, sex, BMI, AST, UA, HDL, eGFR and ABI. Model 3 was the full model and included Model 2, alcohol consumption, and smoking status. Considering the previous literature reporting a possible association between IR and fatty liver, fatty liver was not included as variable for model adjustment. Still, a subgroup analysis was done to find out what the link was between fatty liver, METS-IR, and baPWV. Interaction and stratified analyses were carried out according to age (< 65 and ≥ 65 years), sex (male and female), BMI (< 25, 25–29.9 and ≥ 30 kg/m2), eGFR (≥ 60 and < 60 ml/min), alcohol consumption, smoking status, exercise, fatty liver and hypertension. Stratified linear regression models were used for subgroup analysis.

To explore the nonlinearity of METS-IR and baPWV, we plotted a smooth curve fit (penalized spline approach). We performed a sensitivity study to make sure the data analysis was robust. We computed the P for trend using the METS-IR as a categorical variable. All the analyses were performed with the statistical software packages R 4.0.2 (http://www.R-project.org, The R Foundation) and Free Statistics software versions 1.7. P values less than 0.05 (bilateral) were considered statistically significant.

## 3 Results

### 3.1 Baseline characteristics of the participants

Between March 2004 and December 2012, the clinical characteristics of the medical records of 1445 participants in these health examination programs at “Murakami Memorial Hospital” (897 men and 554 women) were evaluated. 912 people in total participated in this study (592 men and 320 women). In previous work by Fukuda et al., the process of inclusion and exclusion was described in great detail (19).

The baseline characteristics of the participants were showed in **Table 1** based on the METS-IR index quartiles. The average participant age was 51.1± 9.6 years. Significant statistically differences were detected in sex, age, BMI, SBP, DBP, AST, ALT, GGT, FPG, UA, TG, HDL-c, LDL-c, alcohol consumption, smoking status, habit of exercise and fatty liver among the four METS-IR index subgroups (p values < 0.05). Male participants with high alcohol consumption, smoking, and a fatty liver had significantly higher METS-IR. In the exercise habit, the opposite patterns were observed. METS-IR was significantly lower in participants who exercised on a regular basis. This is coherent with our common knowledge and previous research (28).

**Table 1:** Baseline characteristic of the study population according to METS-IR.

Variables	Total (n = 912)	Q1 (n = 228)	Q2 (n = 228)	Q3 (n = 228)	Q4 (n = 228)	*P* value
Sex, n (%)						< 0.001
Male	592 (64.9)	85 (37.3)	127 (55.7)	183 (80.3)	197 (86.4)	
Female	320 (35.1)	143 (62.7)	101 (44.3)	45 (19.7)	31 (13.6)	
Age, (years)	51.1 ± 9.6	50.8 ± 10.1	52.7 ± 9.2	51.3 ± 9.4	49.8 ± 9.4	0.015
BMI, (kg/m2)	23.1 ± 3.1	19.8 ± 1.4	22.3 ± 1.3	23.8 ± 1.5	26.6 ± 2.9	< 0.001
SBP, (mmHg)	120.2 ± 15.0	112.2 ± 13.0	118.1 ± 14.6	123.3 ± 14.4	127.3 ± 13.4	< 0.001
DBP, (mmHg)	76.1 ± 10.0	70.2 ± 8.9	74.6 ± 9.6	78.5 ± 9.5	81.3 ± 8.4	< 0.001
AST, (IU/L)	20.9 ± 8.1	19.4 ± 6.0	19.1 ± 5.8	21.0 ± 6.9	23.9 ± 11.4	< 0.001
ALT, (IU/L)	19.0 (14.0, 26.0)	15.5 (12.0, 19.0)	16.0 (13.0, 21.0)	20.0 (16.0, 27.0)	26.0 (19.8, 40.0)	< 0.001
GGT, (IU/L)	19.0 (14.0, 28.0)	14.0 (11.0, 18.2)	16.0 (13.0, 23.2)	20.5 (15.0, 31.2)	25.0 (18.8, 38.2)	< 0.001
FPG, (mg/dl)	98.1 ± 14.1	92.6 ± 8.0	95.7 ± 9.7	99.2 ± 18.3	104.7 ± 14.8	< 0.001
UA, (mg/dl)	5.2 (4.2, 6.1)	4.4 (3.7, 5.2)	5.0 (4.0, 5.8)	5.7 (4.7, 6.3)	6.0 (5.3, 6.9)	< 0.001
TC, (mg/dl)	209.8 ± 36.0	207.4 ± 38.1	206.5 ± 32.4	209.5 ± 35.2	215.9 ± 37.4	0.025
TG, (mg/dl)	81.0(53.0, 124.0)	50.0 (37.0, 69.2)	65.0 (51.0, 88.0)	92.0 (70.0, 129.2)	142.0 (100.0, 196.2)	< 0.001
HDL-c, (mg/dl)	53.5 ± 14.6	66.6 ± 13.8	57.5 ± 11.4	48.4 ± 9.7	41.7 ± 9.1	< 0.001
LDL-c, (mg/dl)	128.1 ± 31.7	118.3 ± 32.8	125.6 ± 28.0	132.6 ± 31.1	135.7 ± 31.9	< 0.001
eGFR, (mL/min/1.73 m2)	70.4 ± 12.0	74.5 ± 13.3	69.6 ± 11.1	69.1 ± 11.0	68.5 ± 11.7	< 0.001
ABI	56.0 ± 277.6	48.0 ± 265.2	41.7 ± 228.6	61.6 ± 301.8	72.8 ± 308.3	0.631
Alcohol consumption, n (%)						0.006
None or minimal	581 (64.6)	168 (74)	145 (63.9)	129 (57.8)	139 (62.6)	
Light	150 (16.7)	33 (14.5)	39 (17.2)	45 (20.2)	33 (14.9)	
Moderate	88 (9.8)	10 (4.4)	28 (12.3)	28 (12.6)	22 (9.9)	
Heavy	80 (8.9)	16 (7)	15 (6.6)	21 (9.4)	28 (12.6)	
Smoking status, n (%)						< 0.001
None or Past	715 (78.4)	192 (84.2)	191 (83.8)	171 (75)	161 (70.6)	
Current	197 (21.6)	36 (15.8)	37 (16.2)	57 (25)	67 (29.4)	
Habit of exercise, n (%)						0.001
No	719 (80.2)	166 (74.1)	170 (76.9)	184 (81.8)	199 (88.1)	
Yes	177 (19.8)	58 (25.9)	51 (23.1)	41 (18.2)	27 (11.9)	
Fatty liver, n (%)						< 0.001
No	646 (70.9)	222 (97.8)	204 (89.5)	154 (67.5)	66 (28.9)	
Yes	265 (29.1)	5 (2.2)	24 (10.5)	74 (32.5)	162 (71.1)	

Data were mean ± SD or median (IQR) for skewed variables or numbers (proportions) for categorical variables.

BMI, body mass index; SBP, systolic blood pressure; DBP, diastolic blood pressure; AST, aspartate aminotransferase; ALT, alanine aminotransferase; GGT, γ-glutamyltranspeptidase; FPG, fasting plasma glucose; UA, uric acid; TC, total cholesterol; TG, triglyceride; HDL-c, high‐density lipoprotein cholesterol; LDL-C, low-density lipoprotein cholesterol; METS-IR, metabolic score for insulin resistance. Q1, Q2, Q3, and Q4 are quartiles of the metabolic score for insulin resistance (METS-IR).

### 3.2 Univariate and multivariate analyses of METS-IR and baPWV

As **Table 2** shows, univariate analysis indicated that METS-IR, sex, age, BMI, SBP, DBP, AST, ALT, GGT, FPG, uric acid, TC, TG, HDL-c, LDL-c, eGFR and fatty liver were related to baPWV (all p < 0.05). The baPWV increased by 4.64cm (95% CI: 2.21,7.06) for per unit increase in METS-IR.

**Table 2:** Results of univariate analysis of baPWV.

Variable	β (95%CI)	*p* value
METS-IR	4.64 (2.21,7.06)	< 0.001
Sex, n (%)	3.11 (1.46,4.77)	< 0.001
Age, (years)	-49.44 (-82.84, -16.05)	0.004
BMI, (kg/m2)	4.95 (-0.17,10.07)	0.058
SBP, (mmHg)	8.43 (7.51,9.35)	< 0.001
DBP, (mmHg)	11.29 (9.87,12.71)	< 0.001
AST, (IU/L)	3.4 (1.43,5.37)	< 0.001
ALT, (IU/L)	1.5 (0.38,2.61)	0.009
GGT, (IU/L)	1.01 (0.36,1.66)	0.002
FPG, (mg/dl)	4.21 (3.1,5.31)	< 0.001
UA, (mg/dl)	22.77 (11.23,34.31)	< 0.001
TC, (mg/dl)	0.71 (0.27,1.16)	0.002
TG, (mg/dl)	0.44 (0.23,0.65)	< 0.001
HDL-c, (mg/dl)	-1.33 (-2.42, -0.23)	0.018
LDL, (mg/dl)	0.66 (0.16,1.17)	0.01
eGFR, (mL/min/1.73 m2)	-6.39 (-7.65, -5.12)	< 0.001
ABI	0.04 (-0.02,0.1)	0.19
Habit of exercise, n (%)	16.65 (-23.27,56.57)	0.413
Fatty liver, n (%)	93.74 (58.98,128.51)	< 0.001
Alcohol consumption	0.07 (-0.06,0.19)	0.284
Smoking status	-0.16 (-39.07,38.75)	0.994

BaPWV, brachial-ankle pulse wave velocity; 95% CI, 95% confidence interval; METS-IR,metabolic score for insulin resistance; BMI, body mass index; SBP, systolic blood pressure; DBP, diastolic blood pressure; AST, aspartate aminotransferase; ALT, alanine aminotransferase; GGT, γ-glutamyltranspeptidase; FPG, fasting plasma glucose; UA, uric.acid; TC, total cholesterol; TG, triglyceride; HDL-c, high‐density lipoprotein cholesterol; LDL-C, low-density lipoprotein cholesterol; eGFR, estimated glomerular filtration rate; ABI, ankle-brachial index.

**Figure 1:**
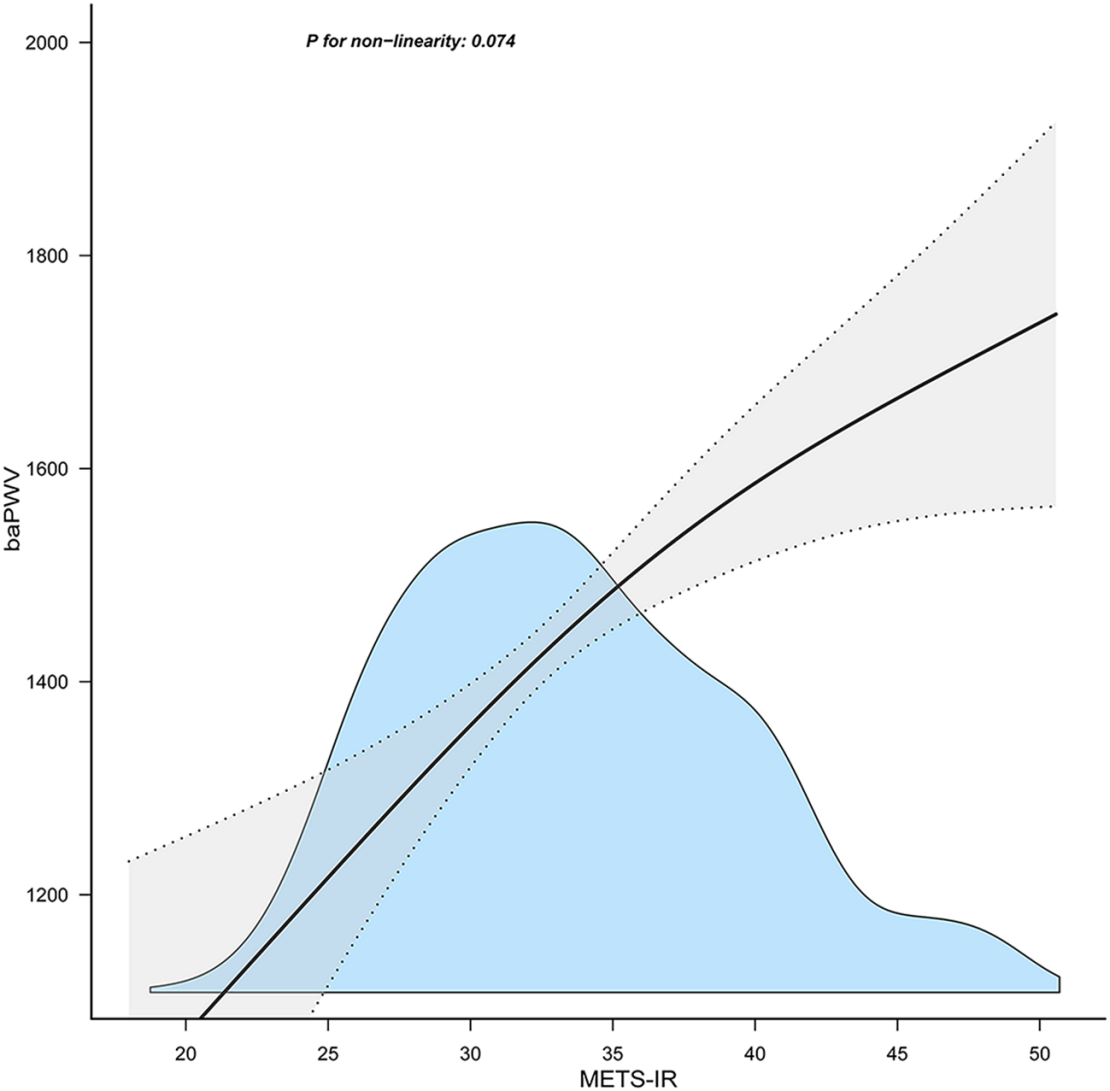
Association between the METS-IR index and baPWV. METS-IR, metabolic score for insulin resistance; baPWV, brachial-ankle pulse wave velocity. Solid and dashed lines represent the predicted value and 95% confidence intervals. They were adjusted for age, sex, body mass index, aspartate aminotransferase, uric acid, high‐density lipoprotein cholesterol, estimated glomerular filtration rate, ankle-brachial index, alcohol consumption, smoking status. Only 99% of the data is shown.

**Table 3** displays the findings of a multivariable regression analysis. After controlling for several variables, METS-IR demonstrated a substantial positive connection with baPWV in all models that were adjusted (adjusted β=15.3, 95% CI: 6.61~23.98, P = 0.001) (**Table 3**). The METS-IR was transformed from a continuous to a categorical variable for sensitivity analysis (quartile). In the fully-adjusted model, the adjusted β of Q2, Q3, and Q4 were 39.64 (95% CI: -8.53~87.8), 86.14 (95% CI:23.45~148.83), and 125.41 (95% CI: 39.99~210.84), with Q1 as the reference point. In addition, the correlation was statistically meaningful across all models (p for trend 0.002). This demonstrated that METS-IR and baPWV are positively correlated. Furthermore, I discovered a positive linear trend between the METS-IR index and the baPWV (**Figure 1**).

**Table 3:** Multivariable-adjust β and 95%CI of the METS-IR index associated with baPWV.

Variable	unadjusted		model 1		model 2		model 3	
	β (95%CI)	*P*_value	β (95%CI)	*P*_value	β (95%CI)	*P*_value	β (95%CI)	*P*_value
METS-IR	4.64 (2.21~7.06)	<0.001	9.44 (4.71~14.17)	<0.001	15.59 (7.11~24.07)	<0.001	15.3 (6.61~23.98)	0.001
1st Quartile(≤28.61)	Ref		Ref		Ref		Ref	
2st Quartile (28.61-32.84)	54.25 (9.5~98.99)	0.018	30.02 (-13.07~73.11)	0.172	34.09 (-13.53~81.71)	0.161	39.64 (-8.53~87.8)	0.107
3st Quartile (32.84-37.62)	85.7 (40.95~130.44)	<0.001	74.47 (23.91~125.02)	0.004	79.68 (18.01~141.35)	0.011	86.14 (23.45~148.83)	0.007
4st Quartile (≥37.62)	97.48 (52.73~142.22)	<0.001	112.9 (47.47~178.33)	0.001	116.89 (32.67~201.11)	0.007	125.41 (39.99~210.84)	0.004
P for trend		<0.001		<0.001		0.004		0.002

Model 1 adjust for age, sex, and BMI.

Model 2 adjust for Model 1+ AST+UA+HDL-c+eGFR +ABI.

Model 3 adjust for Model 1+ Model 2 +alcohol consumption, smoking status.

METS-IR, metabolic score for insulin resistance; baPWV, brachial-ankle pulse wave velocity; 95% CI,95% confidence interval; Ref, reference; BMI, body mass index; AST, aspartate aminotransferase; UA, uric acid; HDL-c, high‐density lipoprotein cholesterol; eGFR, estimated glomerular filtration rate; ABI, ankle-brachial index.

### 3.3 Subgroup analysis

**Figure 2:**
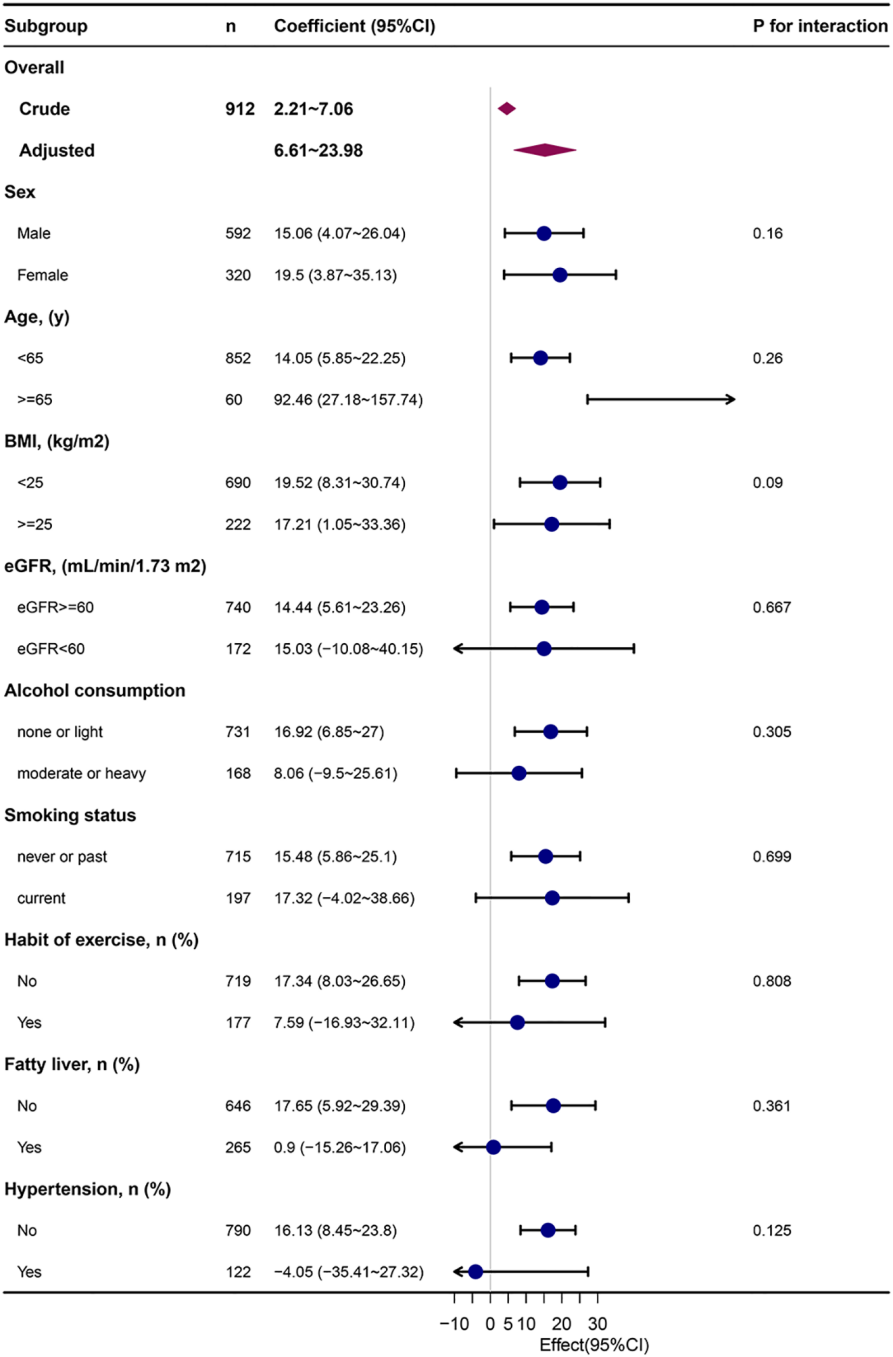
Subgroup analysis of the METS-IR and baPWV. METS-IR, metabolic score for insulin resistance; baPWV, brachial-ankle pulse wave velocity; Each stratification was adjusted for They were adjusted for age, sex, body mass index, aspartate aminotransferase, uric acid, high‐density lipoprotein cholesterol, estimated glomerular filtration rate, ankle-brachial index, alcohol consumption, smoking status except the stratification factor itself. Dots represent effect values, with horizontal lines indicating 95% CIs. Diamonds indicate overall effect values, with outer points of the diamonds indicating 95% CIs.

**Figure 2** depicts the subgroup analysis results. METS-IR was related to baPWV in people with eGFR > 60 ml/min (adjusted β= 14.44, 95% CI: 5.61~23.26, P=0.001), none or light alcohol consumption (adjusted β=16.92, 95% CI: 6.85~27, P=0.001), non-smokers (adjusted β=15.48, 95% CI: 5.86~25.1, P=0.002), non-regular exercisers(adjusted β=17.34, 95% CI: 8.03~26.65, P<0.001), non-fatty liver (adjusted β=17.65, 95% CI: 5.92~29.39, P=0.003), and non-hypertensive (adjusted β=16.13, 95% CI:8.45~23.8, P<0.001).

## 4 Discussion

When controlling for potentially influencing factors, the present cross-sectional study suggests that METS-IR, as both a continuous and categorical variable, is significantly and linearly correlated with arterial stiffness assessed by baPWV in a Japanese medical examination population. The results of the stratified analysis according to the variables including sex, age, BMI, eGFR, alcohol consumption, smoking status, exercise, fatty liver and hypertension were robust and without interaction. The results give evidence that IR contributes to increased arterial stiffness.

The relationship between IR and arterial stiffness has been well recognized. In line with these conclusions, there is becoming increasingly evidence that IR indices that are not based on insulin are linked to arterial stiffness. These indices include TG/HDL-C (15), TyG (16–18), and METS-IR (29–31). Chen Chi et al. (15) found that the correlation between TG/HDL-C and BAPWV is not linear, with an inflection point of 5.6. At TG/HDL-C less than 5.6, TG/HDL-C was positively related to BAPWV [β=12.7, 95% CI (1.9 to 23.5)], while in those who consumed more than 280 g/week of alcohol, TG/HDL-C was inversely linked with BAPWV [β=- 30.7,95% CI (-53.1, -8.4)]. Another cross-sectional study by Lee SB et al. showed that the TyG index was independently associated with increased arterial stiffness in Korean adults, and this association was stronger than HOMA-IR (18). A prospective cohort study of 1895 patients with hypertension or prehypertension over a period of 4.71 years (median follow-up time) similarly discovered a strong correlation between TyG and vascular stiffness progression (32). More recently, Zhang X et al. also found a positive association between TyG and the cardio–ankle vascular index (CAVI) values among Chinese without hypertension over 40 years of age (29). In addition, this association has been validated in Chinese hypertensive patients (16), the Chinese elderly (17), and postmenopausal lean women (33).

METS-IR has been considered in recent years as a novel, reliable, and simple alternative index of IR (12, 28). As far as I know, there are limited studies on METS-IR and arterial stiffness. The cross-sectional study by Roopa Mehta et al. included 358 Mexican DM-free participants and confirmed the association between the HOMA2-IR index, different IR surrogate markers and central aortic stiffness (CAAS) (30). It is thought that using the METS-IR and TyG indices to predict arterial stiffness is highly correlated, easy to figure out, and can be used to measure the risk of heart disease. Bello-Chavolla et al. (31) assessed two main populations: high-risk and non-hypertensive. In their cross-sectional study of a high-risk population (n = 305) with extensive metabolic co-morbidities and dyslipidemia, carotid and femoral pulse waves were used to assess arterial stiffness, and a considerable linear positive connection was found between METS-IR and baPWV in the same way that this study found a significant linear positive correlation between METS-IR and PWV carotid and femoral pulse waves. After adjusting for sex, age, hypertension treatment, and smoking status, multivariate logistic regression analysis suggested that both METS-IR scores (OR 1.03, 95% CI 1.01-1.06) and higher METS-IR scores (OR 2.49 95% CI 1.19-5.23) were associated with PWV >75th percentile and were superior to TG/HDL and TyG metrics. In prospective cohort studies, METS-IR was observed to independently predict hypertensive events, complementing previously validated risk prediction models. Their study adjusted for age, sex, hypertension treatment, and smoking. Our study added to theirs by adjusting for potential confounders such as AST, uric acid, HDL, eGFR, ABI, and alcohol consumption, and the results remained stable. In a recent retrospective study, Zhang X et al. similarly observed a strong correlation between CAVI and non-insulinogenic IR indicators in a non-hypertensive Chinese population. Multivariate logistic regression analysis revealed that the number of people with CAVI 8.0 was much higher in the top quartile of METS-IR than in the bottom quartile (Q4 vs. Q1: OR 2.699, 95% CI 1.235 to 5.897) (29). This accords with the findings of the current investigation. In addition, it has been previously reported in the literature that insulin resistance is stronger in men compared to women (34). However, in this study, stratified analysis was done for gender and the results showed no interaction and the results were robust. Further research may be required.

Different from the above studies assessing arterial stiffness by applying carotid and femoral pulse waves (cfPWV) or CAVI, this study used baPWV to assess arterial stiffness. The baPWV has been reported in the literature to potentially underestimate arterial stiffness in hypertensive patients with a history of cardiovascular events (35). However, baPWV is easier to apply in clinical practice than cfPWV because of its simplicity and ease of measurement (7). Moreover, it has been reported that baPWV closely correlates with directly measured aortic PWV and cfPWV (36, 37). Meta-analyses have also shown that higher levels of baPWV are associated with an increased risk of developing cardiovascular disease (38). That is why baPWV has been used in many studies to assess arterial stiffness. Toshiaki Ohkuma et al. conducted a meta-analysis of 14 673 Japanese subjects without a history of CVD followed for a mean of 6.4 years and the results clearly showed that baPWV was an independent predictor of the risk of developing CVD (Q5 vs Q1, OR 3.50, 95% CI: 2.14-5.74; P<0.001) (8). In addition, most of these aforementioned studies were conducted in populations not at risk for hypertension, non-diabetes, CKD, or CVD, whereas this study complements and extends the relationship between METS-IR and baPWV observed among Japanese health check-up population. By using a simple IR score to evaluate and focus on CVD risk factors, more people may be able to benefit from more aggressive risk factor interventions.

The association between METS-IR, a surrogate marker of IR, and arterial stiffness is supported by pathophysiological mechanisms (2). In IR states, glucose and lipid abnormalities lead to vascular endothelial dysfunction and oxidative stress, both of which activate extracellular matrix metalloproteinases (MMPs), leading to vascular remodeling and arterial stiffness (14). In addition, IR is often accompanied by a chronic inflammatory response, including abnormal production of adipokines, increased release of pro-inflammatory cytokines and chemokines, and infiltration of macrophages and lymphocytes, resulting in vascular endothelial dysfunction and atherosclerosis (39).

Nevertheless, this study had some limitations. First, this study was cross-sectional, so I cannot draw any firm conclusions about cause and effect. Second, since this study uses secondary data from published sources, variables not in the dataset could not be adjusted for, for example, atherosclerosis-related disease history. Third, as with any research study, unmeasured confounders could influence outcomes. Finally, because the participants in this study were all from the Japanese health screening population, it is unclear if the conclusions apply to other ethnicities.

## 5 Conclusions

In conclusion, METS-IR is significantly associated with baPWV among health checkup individuals in Gifu, Japan. As a simple, easily calculated predictor of arterial stiffness, METS-IR could be considered for primary care as a monitoring tool to identify people with high risk of CVD in order to intervene early on risk factors and alleviate atherosclerosis. Future prospective, large-sample studies are still needed to confirm this.

## Data availability statement

The original contributions presented in the study are included in the article/supplementary material. Further inquiries can be directed to the corresponding author.

## Ethics statement

The studies involving humans were approved by The Murakami Memorial Hospital’s ethics committee. The studies were conducted in accordance with the local legislation and institutional requirements. The participants provided their written informed consent to participate in this study.

## Author contributions

GLL: Writing – original draft, Writing – review & editing.
